# Discrepancies between the percentage of plasma cells in bone marrow aspiration and BM biopsy: Impact on the revised IMWG diagnostic criteria of multiple myeloma

**DOI:** 10.1038/bcj.2017.14

**Published:** 2017-02-17

**Authors:** N Lee, S Y Moon, J-h Lee, H-K Park, S-Y Kong, S-M Bang, J H Lee, S-S Yoon, D S Lee

**Affiliations:** 1 Department of Laboratory Medicine, Seoul National University Hospital, Seoul, Korea; 2 Department of Laboratory Medicine, Seoul Clinical Laboratories, Seoul Medical Science Institute, Seoul, Korea; 3 Department of Laboratory Medicine, Center for Diagnostic Oncology, Hospital and Research Institute, National Cancer Center, Goyang, Korea; 4 Department of Internal Medicine, Seoul National University Bundang Hospital, Seongnam, Korea; 5 Department of Internal Medicine, Gacheon University Gil Medical Center, Incheon, Korea; 6 Department of Internal Medicine, Clinical Research Institute, Seoul National University Hospital, Cancer Research Institute, Seoul National University, College of Medicine, Seoul, Korea; 7 Cancer Research Institute, Seoul National University, College of Medicine, Seoul, Korea

Diagnosis of multiple myeloma (MM) is based on diagnostic criteria which include clonal plasma cell (PC) percentage, quantity of monoclonal protein and presence of CRAB symptoms (hypercalcemia, renal insufficiency, anemia, bone lesion).^1, 2^ According to the previously adopted world health organization (WHO) 2008 criteria, the presence of clonal plasma cells is considered as key evidence of clonal plasma cell proliferation, irrespective of the percentage of PC and CRAB features. However, some patients with related organ or tissue impairment symptoms show <10% plasma cells in bone marrow (BM) aspiration. Empirically, there are three indwelling problems with the original criteria utilizing the percentage of plasma cells. Firstly, BM aspiration (BMA) diluted by peripheral blood results in variable plasma cell percentages. At present, no consensus has been made on the ideal diagnostic amount of BM aspirate. Usually, the amount varies from 0.3 to 3 ml depending on the practitioner. Secondly, uneven distribution of plasma cells or patchy infiltration can result in markedly discrepant counts of plasma cells between BMA and biopsy. Thirdly, counting plasma cell % using BM biopsy (BMB) lacks objectiveness, thus making it inapplicable for routine diagnosis.

Recently, international myeloma working group (IMWG) presented revised criteria on plasma cell requirement. In the revised criteria, the percentage of BM plasma cells was emphasized for the diagnosis and discrimination between monoclonal gammopathy of undetermined significance (MGUS) and MM. The most important criterion in diagnosing MM is the presence of clonal plasma cells >10%, whilst a major change from previous criteria is that CRAB features no longer serve as evidence of clonal PC proliferation if bone marrow plasmacytosis is less than 10% and there is no biopsy-proven plasmacytoma.^3, 4, 5^ In addition, to exclude sampling error or patchy bone marrow involvement, IMWG recommend plasma cell % either by aspiration or biopsy, with the higher value being used in cases of discrepancy. This is a modification to the previous version which indicated BMB PC% as non-mandatory.^6^ The percentage of BM PC is also mentioned as a biomarker of malignancy, with a percentage greater than 60% regarded as a myeloma defining event.^7, 8, 9^ Overall, the exact measurement of BM PC is becoming more crucial for the diagnosis of MM, and the introduction of CD138 immunohistochemical stain using BMB has made it possible for more accurate PC counts.^2, 10, 11^ We designed this study to evaluate PC% of BMB in patients with low BMA PC (<10%) and tracked the conversion rate from BMA PC (<10%) to BMB PC (⩾10%).

A total of 389 patients newly diagnosed with MM were enrolled and 67 patients of BMA PC count below 10% were selected. Among 73 cases (67 patients with aspiration PC<10% and 6 patients with unknown PC percentage), 58 were available for analysis of BMB (Figure 1a). For image analysis, CD138 staining using ultraView Universal DAB Detection Kit on BM section was conducted and Image J software, provided by National Institutes of Health (https://imagej.nih.gov/ij/) was utilized. After obtaining the full slide image, the representative image (area cover per spot, 0.96 mm^2^) was selected, and according to the guidelines, the thresholds of hue, saturation, and brightness were adjusted to detect the cytoplasm of plasma cells and nucleus of the other cells (Figure 1b). The total area of plasma cells and the other cells were evaluated separately, and divided by the average size of each cell to count the number of corresponding cells.

From the aforementioned 58 cases, 55 patients demonstrated a BMB PC count of ⩾10%, and only 3 patients showed a BM PC count below 10% (Figure 1a). Mean percentage of PC in BMB was 52.9%, with a standard deviation of 30.7. Interestingly, we observed a bimodal peak in the 20-30% PC percentage group and packed marrow (90-100%) group (Figure 1c). Upon review of BM reports for specimen adequacy, the aspiration quality of only 39.7% of the patients was adequate, and the remaining 60.3% of patients either had mild to severe dilution, or few visible particles. Most of the patients with diluted BMA were found to have an increased PC% on BMB. Patchy infiltration or site variation of plasma cell infiltration was observed in seven patients (Figure 1d).

Clinical features assessed at diagnosis including age, sex, anthropometric laboratory values, and molecular parameters were evaluated by a non-parametric method. The patients were divided into two groups on the basis of BMA PC% (⩾10% or <10%) and the patients with BMA PC<10% were further subdivided into two groups on the basis of BMB PC% (⩾10% or <10%). Creatinine and total protein were statistically significant to be higher in BMA PC⩾10% patients than those of BMA PC<10% patients by Mann-Whitney test, whilst albumin was significantly lower in BMA PC⩾10% patients than that of BMA PC<10% patients (Table 1a).

Additionally, the patients who showed BMA PC% below 10% (a total of 58 patients) were enrolled for investigating the correlation of related clinical parameters. There were moderate positive correlations of β2-microglobulin, BUN, and free light chain (FLC) ratio with BMB PC%, and calcium showed mild correlation with BMB PC%. Correlations of aberrant FISH results among PC% were statistically significant in RB1 deletion, IgH rearrangement, and 1q gain (Table 1b). These patients were also evaluated for prognostic importance. Overall survival (OS) by Kaplan-Meier estimation showed adverse survival rates in high BMB PC% compared to low BMB PC% group (p=0.010). The cutoff value was set as the mean of total BMB PC%, and median OS for high and low BMB PC% group was 93.2 and 67.9 months, respectively (Supplementary Figure 1A). Conversely, BMA PC% showed no significant difference in survival times when analyzed with the same parameters (Supplementary Figure 1B). Univariate Cox analysis showed increased creatinine, BMB PC%, and FLC ratio over 100 to be significantly associated with adverse OS. Among the FISH results, percentage of RB deletion and IgH rearrangement were represented to significant factors (Table 1c).

In the present study, we analyzed the percentage of BMB PC in MM patients with low BMA PC (<10%). Objective measurement of PC% in BM biopsy was performed with BM section stained with CD138, using an image analyzer. The conversion rate of BMA PC <10% patients to >10% in BMB was 75.0% (55/73 patients). Most patients with less than 10% of BMA PC% had diluted BM aspiration and showed diffuse infiltration of plasma cells in BMB, whilst several other patients showed patchy or site varied infiltration.

According to a previous report, about 4% of patients diagnosed with MM showed low plasma cell counts (<10%).^12^ Also, there has been many studies reporting the discrepancy of BMA and BMB.^10, 13^ It would currently be difficult to diagnosis myeloma with only a BMA specimen, thus BMB PC% is expected to help calculate the exact PC amount in each patient. The recently revised IMWG MM criteria also indicate that the higher PC count of either aspiration or biopsy should be considered in cases of discrepancy between specimens. In addition, we found that BMB PC% moderately correlated with corresponding end organ disorders, quantitative FISH results, and overall survival in patients with low BMA PC%. In these patients, BMB PC% better reflected the recently revised criteria rather than BMA PC%. These results were concordant with previous reports that the combined evaluation of bone marrow aspirate and biopsy is more suitable for the prediction of prognosis in multiple myeloma.^14^ Collectively, BMB PC count has more biologic significance in patients with low BMA PC%, and could provide both reliability and diagnostic accuracy in cases with discrepancy.

In conclusion, this is the first in-depth study to have proven that a significant proportion of the low BMA PC% group converted to BMB PC ⩾10% group. In the present study, we tried to overcome the lack of objectiveness for counting BMB PC% by applying image analysis (imageJ software) of CD138+ cells in BM biopsy. Despite previous attempts to count accurate PC% via multicolor flow cytometry^15^ or computer assisted image analysis,^14^ those studies were not practical enough to be incorporated into clinical practice. PC count by image analysis prove to be a practically feasible solution when complemented with automation. In MM, quantification of plasma cells is crucial for diagnosis and prognostication, and the PC count is re-emphasized in the revised IMWG guideline. Since plasma cell counts on BMA or BMB alone harbors the risk of not reflecting the true plasma cell burden, we suggest that the higher value of the PC% among BMB or BMA should be used. In addition, we recommend the potential use of image analysis as a routine tool for objective PC counting in BMB.

## Figures and Tables

**Figure 1:**
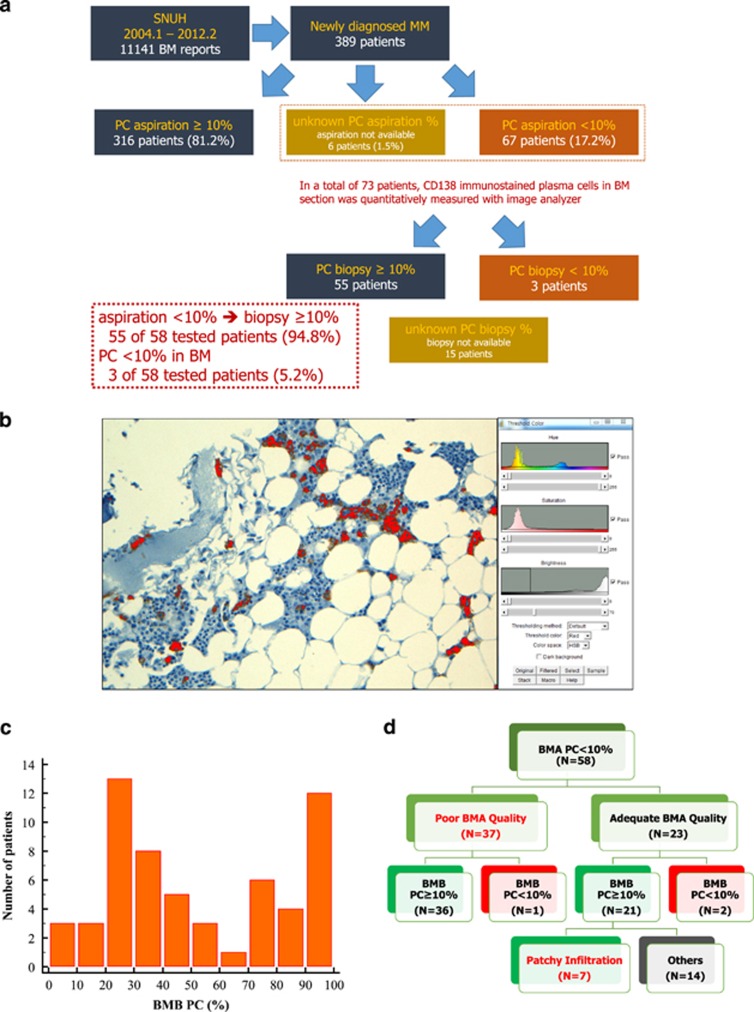
(**a**) Flow chart for patient selection and study design. In a total of 389 newly diagnosed MM patients, 81.2% (316/389) patients had a PC >10%, and the remaining 18.8% (73/389) of patients had either a PC <10% or were unavailable for BMA PC estimation. Of these 73 patients, 55 patients showed PC >10% after estimation by image analysis in bone marrow biopsy. (**b**) Screenshot of plasma cell counting via ImageJ software program. Cytoplasm of plasma cells stained with CD138 is represented in red. Adjustment of the ‘Hue', ‘Saturation', and ‘Brightness' thresholds using ImageJ. (**c**) Distribution of BMB PC percentage among patients with BMA PC less than 10%. When the PC percentage was divided by an interval of 10%, distribution of BMB PC % showed a bimodal peak of 20-30% and 90-100%. (**d**) Classification of patients by bone marrow aspiration quality. Only 39.7% of patients had adequate aspiration quality; the remaining 60.3% of patients had mild to severe dilution. Patchy infiltration/site variation of plasma cell infiltration was shown in 7 patients. MM, multiple myeloma; BM, bone marrow; PC, plasma cell; BMA PC, bone marrow aspiration plasma cell; BMB PC, bone marrow biopsy plasma cell.

**Table 1A:** Demographic and clinical characteristics of the study population

	*BMA PC<10%*^a^		*BMA PC⩾10% (n=316)*	*P value*^a^
	*BMB PC⩾10% (n= 55)*	*BMB PC<10% (n= 3)*		
Age, year	62.3±11.5	60.6±10.1	63±10.3	NS
Male:Female	30:25	2:01	172:144	NS
Hemoglobin (gdl^−1^)	10.2±2.8	8.9±1.8	9.4±2.5	NS
Calcium (mgdl^−1^)	9.0±0.9	8.6±1.5	9.1±1.1	NS
BUN (mgdl^−1^)	21.0±14.0	8.3±2.1	24.2±16.9	NS
Creatinine (mgdl^−1^)	1.5±1.8	0.8±0.2	1.9±1.9	0.002
β2-Microglobulin (mgdl^−1^)	6.0±5.6	4.8±2.1	7.2±6.9	NS
Total Protein (mgdl^−1^)	7.3±1.7	7.4±1.5	8.0±2.1	0.025
Albumin (mgdl^−1^)	3.6±0.7	2.8±0.7	3.4±0.8	0.006
BM aspiration PC%	4.2±3.5	4.1±2.7	39.5±23.4	<0.001
BM section PC%	32.2±17.8	4.0±4.0	N/A	NS

Abbreviations: BMA, bone marrow aspiration; BMB, bone marrow biopsy; PC, plasma cell; CRAB, hypercalcemia, renal insufficiency, anemia, bone lesion; PEP, protein electrophoresis; N/A, not assessed; NS, not significant.

a Values are presented as mean and standard deviation.

Patients were classified into two sub-groups according to bone marrow section plasma cell percentage.

P-value for Mann-Whitney test between group with BMA PC<10% and group with BMA PC⩾10%.

**Table 1B:** Correlation statistics of PC% in BMB with results of FISH and clinical parameters

	*Correlation coefficient (r*^*2*^)	*P-value*
Hemoglobin (gdl^−1^)	−0.254	0.054
Total calcium (mgdl^−1^)	0.304	0.021
BUN	0.683	0.004
Creatinine (mgdl^−1^)	0.342	0.009
FLC ratio (>100 or <0.01)	0.575	<0.001
β2-microglobulin	0.645	0.009

*FISH*		
RB1 deletion (%)	0.306	0.034
IgH rearrangement (%)	0.347	0.016
Trisomy 1 (%)	0.387	0.007

Abbreviations: BMB, bone marrow biopsy; BUN, blood urea nitrogen; FLC, free light chain; PC, plasma cell.

**Table 1C:** Univariate analysis of prognostic factors significantly associated with overall survival among patients (n=58) with BMA PC less than 10%.

*Variables*	*Univariate analysis*		
	*Hazard ratio (HR)*	*95% Confidential Interval (CI)*	*P-value*
Hemoglobin (gdl^−1^)	1.03	0.80-1.31	0.841
Total calcium (mgdl^−1^)	1.41	0.79-2.54	0.249
Creatinine (mgdl^−1^)	1.25	1.06-1.48	0.010
Bone lesion	1.12	0.34-3.70	0.846

*FISH*			
RB1 deletion (%)	1.03	1.00-1.07	0.034
IgH rearrangement (%)	1.04	1.01-1.07	0.010
p16 deletion (%)	0.01	0.00-1117.1	0.963
Trisomy 1 (%)	1.03	1.00-1.07	0.053
BMA PC (%)	0.91	0.74-1.08	0.239
BMB PC (%)	1.04	1.02-1.07	0.002
FLC ratio (>100 vs ⩽100)	11.1	2.22-55.07	0.003

Abbreviations: BMA, bone marrow aspiration; BMB, bone marrow biopsy; FLC, free light chain; PC, plasma cell.
