# Supplementary figures and images for: Discrepancies between the percentage of plasma cells in bone marrow aspiration and BM biopsy: Impact on the revised IMWG diagnostic criteria of multiple myeloma
Source: Blood Cancer J. 2017 Feb 17;7(2):e530–. doi: 10.1038/bcj.2017.14 (PMC5386332; doi:10.1038/bcj.2017.14)

## Slide 1
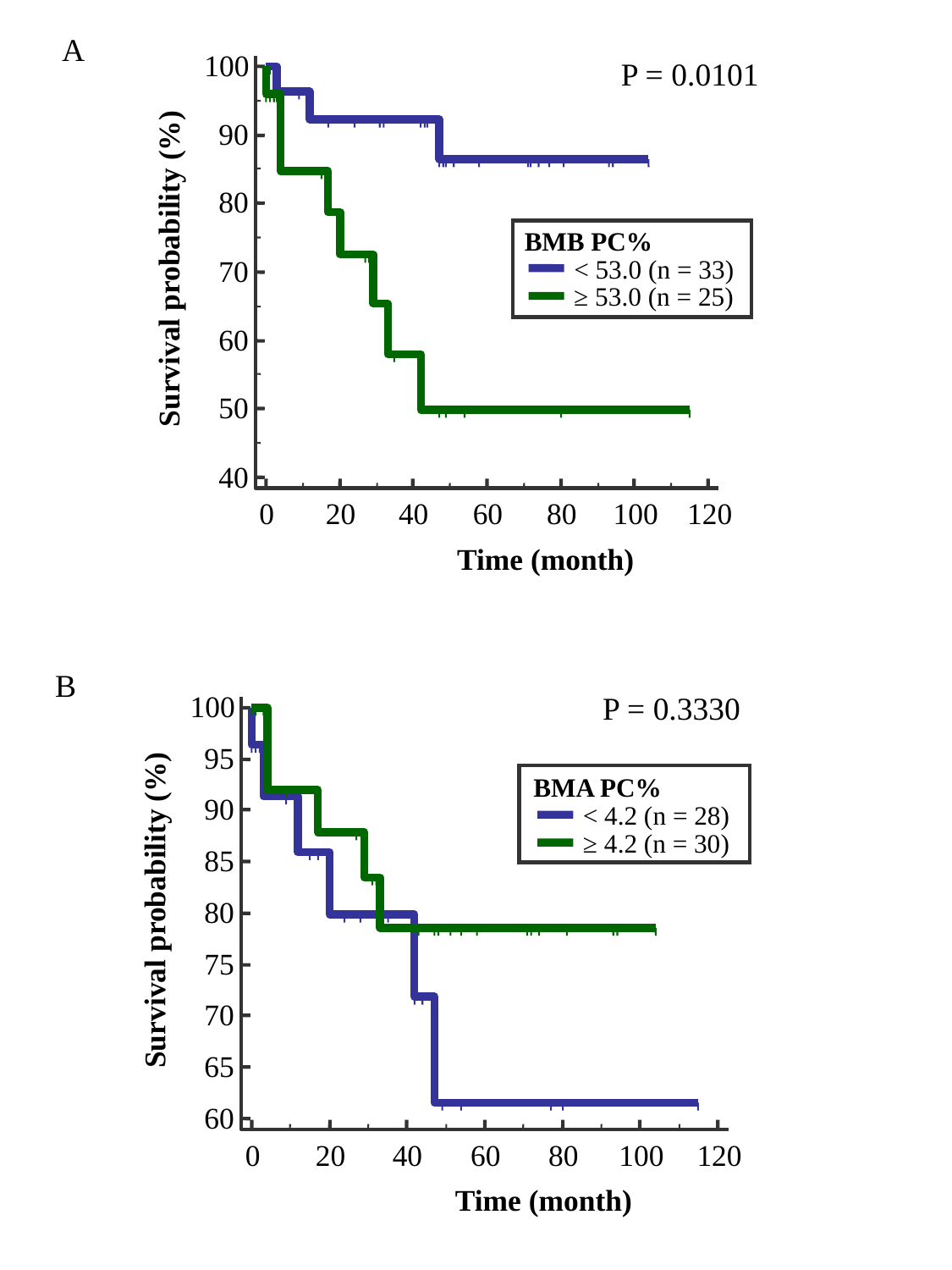

A
100
P = 0.0101
90
80
BMB PC%
Survival probability (%)
70
< 53.0 (n = 33)
≥ 53.0 (n = 25)
60
50
40
0
20
40
60
80
100
120
Time (month)
B
P = 0.3330
100
95
BMA PC%
90
< 4.2 (n = 28)
≥ 4.2 (n = 30)
85
Survival probability (%)
80
75
70
65
60
0
20
40
60
80
100
120
Time (month)

Supplement: Supplementary Figure [file bcj201714x1.ppt]
