# Supplementary material for: Discrepancies between the percentage of plasma cells in bone marrow aspiration and BM biopsy: Impact on the revised IMWG diagnostic criteria of multiple myeloma
Source: Blood Cancer J. 2017 Feb 17;7(2):e530–. doi: 10.1038/bcj.2017.14 (PMC5386332; doi:10.1038/bcj.2017.14)
Supplement: Supplementary Information [file bcj201714x2.docx]

**SUPPLEMENTARY INFORMATION**

**Discrepancies between the percentage of plasma cells in bone marrow (BM) aspiration and BM biopsy: Impact on the revised IMWG diagnostic criteria of multiple myeloma**

**Supplemental Methods**

**Patients**

We studied a total of 389 patients newly diagnosed with Multiple myeloma (MM) at Seoul National University Hospital. Among them, we selected patients with bone marrow (BM) aspiration plasma cell (PC) count was <10%. CD138 staining in BM biopsy was conducted for these patients, and plasma cell counts were analyzed via image analysis. Furthermore, we reviewed the clinical aspects of these patients, by referring to their past medical records. Among a total of 389 patients, 67 patients (17.2%) had a PC cell count less than 10% in BM aspiration. The remaining 316 patients had a PC percentage of ≥10%, whilst it was difficult to count PC in 6 patients due to inadequate specimen or unavailability of BM section in cases of consultations from other hospitals.

Amongst 73 cases (67 patients with aspiration PC < 10% and 6 patients with unknown PC percentage), 58 were available for biopsy image analysis. From these 58 cases, 55 patients demonstrated a BM biopsy PC count of ≥10%, and only 3 patients showed a BM PC count below 10%. The BM samples were collected with informed consent, and the study was reviewed and approved by the Institutional Review Board of Seoul National University College of Medicine.

**CD138 staining and image analysis**

The overall cellularity and PC% were counted using BM biopsy sections. According to standard instructions, paraffin-embedded samples were decalcified by 10% neutral-buffered formalin (Australian Biostain, Pty. Ltd., Traralgon, Australia). Thin-layer sections were stained with hematoxylin and eosin (H&E) and immunohistochemically for CD138 using ultraView Universal DAB Detection Kit (Ventana Medical Systems Inc., Tucson, AZ, USA) on Ventana Benchmark XT platform, following the manufacturer's protocols. The slides were immersed in citrate buffer and were dried in a microwave for antigen retrieval. The slides were pretreated with a EDTA-buffered cell conditioning 1 solution (CC1, Ventana Medical Systems Inc.) for 24 min, and incubated with monoclonal antibody CD138 (clone QBEnd10, Novocastra, Leica Biosystems, Newcastle upon Tyne, UK) for 42 ℃, 16 min. The slides were then counterstained, and mounted. BMB PC was estimated by slide scanned image and image analyzer. The bone marrow biopsy slide was fully scanned by ImageScope software, version 10.2.1.2314 (Aperio Technologies, Vista, CA). Theses slide images were analyzed using Image J software, provided by National Institutes of Health (https://imagej.nih.gov/ij/). After obtaining the full slide image, the representative image (area cover per spot, 0.96 mm^2^) was selected, and following the guidelines, the thresholds of hue, saturation, and brightness were adjusted to detect the cytoplasm of plasma cells and nucleus of the other cells (exclude plasma cells). The total area of plasma cells and the other cells were evaluated separately, and divided by the average size of each cell to count the number of corresponding cells. BM biopsy PC% was calculated by the equation: BM biopsy PC% = (Number of PC) / (Number of PC + Number of other cells) x100.

**Fluorescence in situ hybridization (FISH)**

Interphase FISH was performed on patients’ BM aspiration specimens and cultured BM stromal cells to investigate common chromosomal abnormalities known to frequently occur in MM. Commercial FISH probes included an LSI dual-color, break-apart probe for *IGH* translocations; a dual color, dual-fusion translocation probe for t(14;16)(q13;q32)/*IGH-MAF*; a dual color, dual-fusion translocation probe for t(4;14)(p16;q32) /*IGH-FGFR3*; a LSI 13 (*RB1*) 13q14 probe;an LSI p53 (17p13.1) probe; a Vysis LSI p16 (9p21) SpectrumOrange/CEP 9 SpectrumGreen probe; and an LSI 1p36/1q25 probe (all from Abbott Molecular/Vysis, Des Plaines, IL). Plasma cells in BM aspirates were tested individually using a modification of the simultaneous κ/λ immunoglobulin light chain cytoplasmic staining method (cIg FISH) following the manufacturer’s procedure. Five microliters of a 1:20 dilution of a 1:1 mixture of polyclonal anti-human κ (Clone F0198; DakoCytomation, Glostrup, Denmark) and anti-human λ (Clone F0199; Dako) was added to the samples, which were then incubated for 40 minutes in the dark in a humidified chamber. Slides were washed twice in phosphate-buffered saline (PBS) and dried. Slides were stained with FISH probes and counter-stained with DAPI, and then, fluorescence signals were analyzed with a fluorescence microscope (Zeiss, Göttingen, Germany). Interphase FISH signals were evaluated in 20 plasma cells. The results of the FISH studies were recorded according to the ISCN 2013.

**Statistical analysis**

Clinical features assessed at diagnosis including age, sex, anthropometric laboratory values, and molecular parameters were evaluated by a non-parametric method. The patients were divided into two groups on the basis of bone marrow aspiration (BMA) PC% (≥10% or <10%) and the patients with BMA PC<10% were further subdivided into two groups on the basis of bone marrow biopsy (BMB) PC% (≥10% or <10%). Mann-Whitney test was performed to clarify the statistical importance in clinical parameters and FISH results. Also, correlations between percentage of plasma cells in BMB section and laboratory parameters for related end organ diseases were studied. Cumulative OS curves for each group were calculated by using Kaplan–Meier method and were compared by using log-rank test. Prognostic impact of BMB PC on OS was assessed by using Cox proportional hazard model. Statistical significant value was set at p-value of <0.05. All statistical analyses were performed by using MedCalc for Windows, version 12.5 (MedCalc Software, Ostend, Belgium)

**Supplementary Figure legend**

**Supplementary Figure 1.** Kaplan-Meier survival analysis are shown according to the mean percentage of plasma cells (A) in bone marrow biopsy (mean, 53.0%), (B) in bone marrow aspiration (mean, 4.2%). A total of 58 patients who showed plasma cells in bone marrow aspiration below 10% were enrolled.
